# AGING AND NUMERACY: A META-ANALYSIS

**DOI:** 10.1093/geroni/igad104.1539

**Published:** 2023-12-21

**Authors:** Ryan Best, Farima Sadeqi

**Affiliations:** West Virginia University, Morgantown, West Virginia, United States; West Virginia University, Morgantown, West Virginia, United States

## Abstract

Numeracy, the ability to competently make use of numbers and numerical information, is a unique cognitive ability associated with diverse positive outcomes across the lifespan. Often included as a covariate in studies of cognitive aging, there is some scattered evidence of lower numeracy in older adults when compared to younger adults. Importantly, one longitudinal study found evidence of a quadratic decline in numeracy starting in later middle-age. The current study systematically reviews the literature on aging and numeracy as measured by one of three frequently implemented objective measures: the Lipkus numeracy scale (Lipkus et al., 2001), Weller et al.’s Rasch-based numeracy scale (2013), and the Berlin Numeracy Test (Cokely et al., 2012). Effect sizes were calculated from two types of studies: those including age as a categorical extreme group comparison variable or as a continuous variable from adult lifespan samples. Hedge’s g was computed as the effect size in the former studies and Pearson’s r was calculated as the effect size for the latter. For comparison across studies in a single random-effects model, Pearson’s r effect sizes were mathematically transformed into Hedge’s g’s. Type of sample (extreme group vs. adult lifespan) and age range were included in a second mixed-effects model to account for heterogeneity in effect sizes. Initial results show lower numeracy in older adults, but reduced effect size estimates in lifespan samples compared to those using extreme group comparisons. The implications for using linear estimates of effect size for the quadratic relationship between age and numeracy are discussed.

